# Effects of Provenance, Growing Site, and Growth on *Quercus robur* Wood Anatomy and Density in a 12-Year-Old Provenance Trial

**DOI:** 10.3389/fpls.2022.795941

**Published:** 2022-04-29

**Authors:** Peter Hietz, Kanin Rungwattana, Susanne Scheffknecht, Jan-Peter George

**Affiliations:** ^1^Department of Integrative Biology and Biodiversity Research, Institute of Botany, University of Natural Resources and Life Sciences, Vienna, Austria; ^2^Department of Botany, Faculty of Science, Kasetsart University, Bangkok, Thailand; ^3^Department of Forest Genetics, Federal Research and Training Centre for Forests, Natural Hazards and Landscape, Vienna, Austria; ^4^Faculty of Science and Technology, University of Tartu, Tartu, Estonia

**Keywords:** provenance trial, *Quercus robur*, wood anatomical traits, climate effect, vessel size distribution

## Abstract

Vessels are responsible for an efficient and safe water transport in angiosperm xylem. Whereas large vessels efficiently conduct the bulk of water, small vessels might be important under drought stress or after winter when large vessels are embolized. Wood anatomy can adjust to the environment by plastic adaptation, but is also modified by genetic selection, which can be driven by climate or other factors. To distinguish between plastic and genetic components on wood anatomy, we used a *Quercus robur* trial where trees from ten Central European provenances were planted in three locations in Austria along a rainfall gradient. Because wood anatomy also adjusts to tree size and in ring-porous species, the vessel size depends on the amount of latewood and thereby ring width, we included tree size and ring width in the analysis. We found that the trees’ provenance had a significant effect on average vessel area (VA), theoretical specific hydraulic conductivity (Ks), and the vessel fraction (VF), but correlations with annual rainfall of provenances were at best weak. The trial site had a strong effect on growth (ring width, RW), which increased from the driest to the wettest site and wood density (WD), which increased from wet to dry sites. Significant site x provenance interactions were seen only for WD. Surprisingly, the drier site had higher VA, higher VF, and higher Ks. This, however, is mainly a result of greater RW and thus a greater proportion of latewood in the wetter forest. The average size of vessels > 70 μm diameter increased with rainfall. We argue that Ks, which is measured per cross-sectional area, is not an ideal parameter to compare the capacity of ring-porous trees to supply leaves with water. Small vessels (<70 μm) on average contributed only 1.4% to Ks, and we found no evidence that their number or size was adaptive to aridity. RW and tree size had strong effect on all vessel parameters, likely *via* the greater proportion of latewood in wide rings. This should be accounted for when searching for wood anatomical adaptations to the environment.

## Introduction

Wood is responsible for the mechanical stability of trees, for water transport, and for storage and production of metabolites (Hacke and Sperry, 2001; Chave et al., 2009; Morris et al., 2016). In angiosperms, these functions are mainly mediated by fibers, vessels, and living axial and radial parenchyma, respectively. To ensure that a sufficient amount of water is supplied to the transpiring leaves, vessels need to transport the water efficiently. This requires a sufficiently high hydraulic conductance, which is mainly achieved by large vessels and to a lesser degree by vessel density (Tyree and Zimmermann, 2002). At the same time, water transport under high tension needs to avoid the formation of gas emboli and the consequent blockage of the water transport pathway (Tyree and Sperry, 1989). While the anatomical basis for efficient water transport is mainly wide vessel lumina but also the resistance of the cell walls to water passage (Sperry et al., 2005), embolism resistance is mainly a function of the fine structure of the cell walls and particularly the pit membranes connecting adjacent vessels (Delzon et al., 2010; Lens et al., 2011). Although frequently proposed, at least at the inter-specific level, the trade-off between efficiency and safety of plant water transport is quite weak as shown by a global review of data (Gleason et al., 2015). According to the air-seeding hypothesis (Tyree and Sperry, 1989) of embolism formation under drought stress, which is the model best supported by experimental evidence, vessels will embolize when the pressure difference between a gas-filled and a water-filled element is high enough to suck gas through the largest pore in the pit membrane. As the size of these pores is quite variable, the larger vessels with more pit membranes will likely have a larger maximum pore size and thus cavitate at a higher water potential (rare pit hypothesis, Christman et al., 2009, 2012). Therefore, within one species or stem, larger vessels tend to embolize earlier (Jacobsen et al., 2019; Lemaire et al., 2021) and wood with smaller vessels should be more cavitation resistant.

Emboli can are also be formed by freeze-thaw events (Mayr and Améglio, 2016). Gas is hardly soluble in ice and forms bubbles when water freezes. Upon thawing, these bubbles may either dissolve in water, or coalesce and expand, resulting in emboli. The formation of freezing-induced emboli is more likely if water potential is low, which expands the bubbles, and when vessels are large, in which case many bubbles are released that can coalesce into larger ones that will not easily dissolve. As a consequence, species with larger conduit diameters will become more easily embolized following freezing under moderate drought stress. Experiments confirmed predictions that cavitations will occur in vessels with a diameter greater than c. 44 μm under a moderate xylem tension of 0.5 MPa (Davis et al., 1999). Thus, ring-porous trees that have their earlywood macropores embolized after winter and cannot reverse the emboli need to produce new wood before water can be transported to the new leaves (Hacke and Sauter, 1996).

Tree ring research and dendroclimatology mostly rely on ring width, often separating early- and latewood (Schweingruber, 2007). Wood anatomical parameters may yield additional information on past climates or the impact of climate as cell size and numbers might respond more flexibly to variation in temperature and water supply. For instance, conifer ray parenchyma was shown to carry a rainfall signal, although the correlation among trees and the signal-to-noise ratio of the parenchyma was much lower than for ring width (Olano et al., 2013).

More commonly than parenchyma, vessel size and density are measured to support tree ring research (Fonti et al., 2010; Souto-Herrero et al., 2017) and to study hydraulic adaptations of trees (Hietz et al., 2017; Gouveia Fontes et al., 2020). Large vessels of macroporous and ring-porous temperate trees are attractive to study because they are relatively easy to measure in an automated way (Fonti et al., 2009) and they also account for the bulk of water transport capacity. Thus, large vessels (defined as > 10,000 μm^2^) were found to provide an additional signal for spring precipitation in *Quercus petraea* and *Q. pubescens* in Switzerland (Fonti and García-González, 2008). Another study, using a vessel size threshold of >2,500 μm^2^, found that vessel size was related to the previous year wet season in *Quercus boissieri* but to the current wet season in in *Q. ithaburensis* (Castagneri et al., 2017).

It is important to note, however, that short-term adjustments to drought or the effects of drought periods on wood formation are not necessarily the same as growing in arid regions. While inter-annual variation in wood structure is the basis of dendroclimatology, it does not provide a perfect prediction of the effect of long-term changes in climate or of how a genotype would fare in a different environment. Other studies therefore looked at vessel size in trees growing in different environments or originating from different regions and growing in the same environment to identify phenotypic (plastic) and genetic adaptations. For instance, a provenance trial of rubber trees (*Hevea brasiliensis*) found that vessel density and vessel lumen fraction (the proportion of the cross-sectional area covered by vessel lumina) were related to the amount of rainfall in the dry season from the region in Brazil where the genotypes were collected, but vessel size was not (Rungwattana et al., 2018). While that study tested for genetic adaptations, variation observed between trees growing in different environments may result from genetic and/or phenotypic variation. Thus, the significant correlation of *Fagus sylvatica* vessel size with mean annual precipitation at five sites (Schuldt et al., 2016) may have resulted from genetic or phenotypic effects or both.

If large vessels are responsible for the bulk of hydraulic conductance, particularly in ring-porous species such as *Quercus* with very large differences in vessel size, this raises the question what small vessels are for. With a trade-off between efficiency and vulnerability at the level of individual vessels, micropores could provide for a minimum conductance when large vessels have become embolized. If this is indeed a relevant function of micropores, we would expect to see a higher proportion of small vessels in trees adapted to lower water availability. However, with macropores mainly in earlywood and micropores mainly in latewood, the mean vessel size and the vessel size distribution will also depend on the relative areas of early- and latewood and thus on ring width. Therefore, growth rates, which are strongly affected by factors such as water supply, may blur possible adaptive changes in vessel sizes unless the effects of growth rate or ring width are accounted for.

We here used a *Quercus robur* trial with trees from ten provenances from central Europe planted at three sites in Austria. A previous analysis focusing on growth found strong local adaptation in growth so that trees grow best in climates similar to those the seeds were collected from George et al. (2020). In that study, we found that wood anatomical parameters were more controlled by provenance than site, but there was no clear correlation with climate at the provenance location. However, that study did not explore vessel size variation and the relationship with tree size and growth. Having manually marked large as well as small vessels, we here test to what extent vessel size and vessel size distribution are controlled by genetics and environment. We ask how much small vessels likely contribute to water transport and if small vessels and vessel size distribution reflect genetic and plastic adaptations and tree size. If wood anatomical variation can be explained by variations of tree size and growth rates alone, this would question the common interpretation as evidence adaptive significance.

## Materials and Methods

*Quercus robur* seedlings of 20 provenances from Austria, Czech Republic, Croatia, and Slovenia were planted at three trial sites in Austria (Table 1) in 2006. Trees were planted as 1-year-old seedlings with a distance of 1 m × 2 m on flat terrain. We selected ten provenances that differ mainly in rainfall and less in temperature (Table 1) because the purpose of our study was to look for the effects of aridity. Similarly, trial sites differed mainly in rainfall with mean annual precipitation for the three trial sites (MAPs, 641, 890, and 1,005 mm, respectively), similar to the precipitation gradient, the provenances were collected from (MAPp). By contrast, mean annual temperature varied less among provenances (MATp) and very little among trial sites (MATs). MAT and MAP for provenances and testing sites were derived from the EURO-CORDEX climate dataset (Jacob et al., 2014). For each provenance and site, 110 trees were established with 5 half-siblings from each of 22 mother trees planted in a randomized block design with each provenance replicated three times per site and with each mother tree represented five times in each cell [see George et al. (2020) for details]. We randomly selected trees from nine different mother trees from each of the ten provenances at each site (90 trees per site). Trees originating from the same mother trees (half-siblings) were replicated among, but not within sites.

**TABLE 1:** Provenances in central Europe and trial sites in Austria for a *Quercus robur* study.

	Code	Country	Latitude (°N)	Longitude (°E)	MAT (°C)	MAP (mm/year)
**Provenance**						
Geinberg	1	AT	48.277	13.307	8.7	1066
Linz	2	AT	48.326	14.294	9.2	841
Braunsberger Wald	6	AT	48.473	16.333	9.1	638
Rainfeld	8	AT	48.042	15.732	7.4	785
Luising	12	AT	47.023	16.477	9.7	696
Klagenfurt	14	AT	46.630	14.350	8.0	988
Hluboka	17	CZ	49.090	14.444	7.4	764
Kutina	18	HR	45.433	16.683	10.9	915
Murska suma	19	SLO	46.498	16.511	10.1	810
Velika Gorica	21	HR	45.674	16.161	10.5	920
**Trial sites**						
Weyerburg	Dry	AT	48.557	16.171	8.8	641
Wels	Interm.	AT	48.185	13.989	8.5	890
Weistrach	Wet	AT	48.053	14.563	8.4	1005

In winter 2017–18, wood samples were collected at breast height, taking care to avoid irregular growth, from nine trees per provenance and site using a 5.15-mm diameter increment borer. Samples covering at least the last 3 years were sealed in plastic straws in the field and measured for wood density (WD) in the laboratory within 30 h.

**FIGURE 1:**
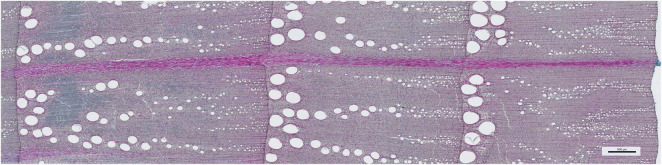
*Quercus robur* wood (the example is from a tree from provenance Geinberg growing at site Wels) showing growth rings for 2015–2017. The scale bar measures 500 μm.

Wood density of each sample was calculated as dry weight (100°C)/fresh volume, with volume calculated from the diameter (5.15 mm) and the length of the sample, the inner end of which was trimmed with a sharp blade, the outer end being the cambium. We prefer this method of measuring the volume to the water displacement method because with the latter, the volume can be affected by variable swelling of wood after cores are extracted (Schüller et al., 2013). We made 30-μm transverse sections covering the width of the core and the last three growth rings with a rotary microtome (Leica, Wetzlar, Germany), stained with safranin and astrablue, and embedded in Euparal (Carl Roth, Karlsruhe, Germany). Digital images (example in Figure 1) with pixel size of 1.157 μm were taken with a DM5500B microscope (Leica, Wetzlar, Germany). The outline of the three outer growth rings (representing years 2015–2017) and all vessels were marked manually in Adobe Photoshop CS6 (Adobe Systems, San José, USA), using the flood-fill feature of Photoshop to mark vessels. Marking vessels manually is more time-consuming than automated vessel detection based on the cell size and shape (e.g., Von Arx and Dietz, 2005), but we found this more reliable to distinguish small vessels from parenchyma. The marked vessels and the growth ring outlines were then measured automatically with ImageJ,^1^ so that all vessels were allocated to the precise year. Ring width was measured as the distance between ring boundaries along the rays. We calculated mean vessel area (VA, mm^2^), vessel density (VD, vessels mm^–2^), vessel lumen fraction (VF, the sum of vessel lumina per cross-sectional area), the coefficient of variation of vessel size (Vcv), and the mean area macropores (VAmp, defined as vessels with diameters > 70 μm). Since large vessels are more efficient in water transport while small vessels may be more resistant against emboli (Jacobsen et al., 2019), vessel size variation would indicate how these two demands shape vessel sizes.

### Data Analysis

We calculated the theoretical specific hydraulic conductivity (Ks), based on the Hagen–Poiseuille law (Tyree and Zimmermann, 2002) as Ks = (πρw/128 η) × VD × Dh^4^, where ρw is the density of water (998.2 kg/m^3^ at 20°C), η the viscosity of water (1.002 × 10^–3^ Pa s at 20°C), VD vessel density (m^–2^), and Dh = (Σ D^4^/n)^1/4^, where D is the average of minor and major axes of the diameter of each individual vessels and n the number of measured vessels. All anatomical parameters were calculated per sample and per annual ring for each sample, and these sample or annual ring-wise data were used for statistical analyses.

**FIGURE 2:**
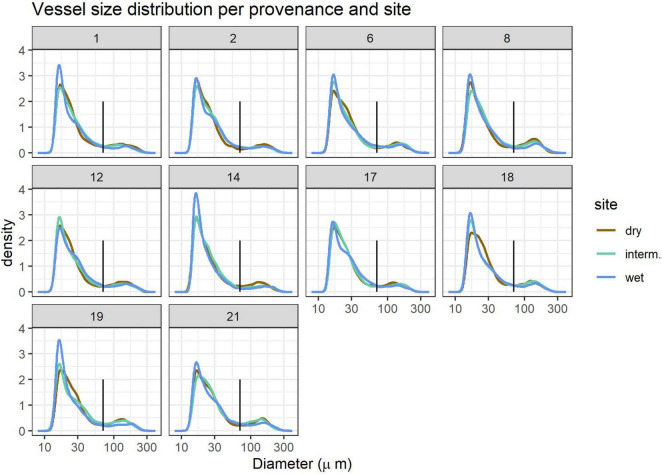
Vessel size distribution per provenance 1–21 (panels) and site (line colors). The vertical black line indicates the 70 μm size cutoff used to define small and large vessels. Kernel density estimates are relative units. Site details are shown in Table 1.

Vessel size distribution showed a bi-modal distribution with the lowest density for vessels with 70 μm diameter, which held true for all provenances and sites (Figure 2). We thus defined vessels < 70 μm as micropores and vessels > 70 μm as macropores and calculated the fraction of vessel area occupied by macropores (p_macro_) and the proportion of Ks contributed by micropores (Ks_micro_). While large vessels are concentrated in earlywood and small vessels in latewood in most ring-porous trees such as *Q. robur*, this is not always the case and we therefore do not attempt to classify early- and latewood.

We then calculated pairwise correlations between all wood traits. To assess the coordination among wood traits and the dominant trait axes, we calculated a principal component analysis (PCA, using R libraries prcomp for calculation and factoextra for visualization) of traits and provenance climate with trait values per sample scaled to mean = 0 and unit variance.

Trees widen conduits to adjust to increasing path length (Anfodillo et al., 2006), so tree height might be a better predictor of conduit size than diameter. Since height was measured only in 1 year and correlates strongly with diameter at breast height (DBH, Supplementary Figure 1), we use DBH as a measure of tree size. We calculated linear models to test whether wood anatomical traits were controlled by provenance, site, provenance × site interactions, or tree size of the form


(1) $${\mathrm{trait}}_{\mathrm{ijk}}∼{\mathrm{provenance}}_{j}\times {\mathrm{site}}_{k}+{\mathrm{DBH}}_{\mathrm{ijk}}+\varepsilon_{\text{ijk}}$$

where “trait_ijk_” is the trait value per tree i (i.e., covering all years analyzed) from provenance j at site k and ε is the error term. Since wood anatomy frequently changes as trees grow or from pith to cambium in wood cores (Lachenbruch et al., 2011; Rungwattana and Hietz, 2018) and trees had grown at different rates, DBH was included in the model, so that potential differences in wood anatomy among sites or provenances would be independent of trees having grown at different rates. While this tests for the effect of provenance, it does not test whether the provenance effect is caused by differences in MAPp, and we therefore calculated a similar model with MAPp instead of provenance with models of the form


(2) $${\mathrm{trait}}_{\mathrm{ijk}}∼{\mathrm{MAPp}}_{j}\times {\mathrm{site}}_{k}+{\mathrm{DBH}}_{\mathrm{ijk}}+\varepsilon_{\text{ijk}}$$

where MAPp_j_ is the MAP at provenance j. To test whether diameter increment or precipitation in any year affect wood traits, we calculated mixed effect models (R library lme4, Bates and Sarkar, 2020) with site, provenance, DBH, ring width, and precipitation (prec) during the growth year at the three sites as fixed and treeID and year nested in site as random variables. Models were thus of the form:


(3) $$\begin{array}{c} {\mathrm{trait}}_{\mathrm{ijky}}∼{\mathrm{provenance}}_{j}\times {\mathrm{site}}_{k}+{\mathrm{prec}}_{\mathrm{ky}}+\mathrm{ring}{\mathrm{width}}_{\mathrm{ijky}} \end{array}$$

where “trait_ijky_” is the value per tree i from provenance j at site k and annual growth ring y, prec_ky_ is the precipitation at site k in the year y, and ring width and DBH are also per year y. For the annual model, DBH for 2016 was estimated as DBH − 2 × ring width of 2017 and DBH_2015_ likewise by subtracting growth of 2016 and 2017. TreeID was treated as a random variable because there were three measurements per tree, and precipitation to avoid pseudo-replication of precipitation, for which data could only be included for three sites and 3 years. While ring width and DBH were correlated, the correlation was not very high (*r*^2^ = 0.27) and the variance inflation factor for ring width and DBH is <2, so the collinearity is not a problem for the model, and we can distinguish the effects of tree size and ring width. Precipitation for 2015, 2016, and 2017 was the total rainfall from October of the previous to September of the current year. Precipitation for the three trial sites was obtained from the closest weather station by the Zentralanstalt für Meteorologie und Geodynamik.^2^

VA, VD, VF, Ks, p_macro_, and Ks_micro_ were log-transformed for statistical analyses to improve normality of the distribution based on the qqnorm plots. All statistical analyses were calculated with R 4.0.2 (R Core Team, 2020).

## Results

Vessel size distribution followed a similar pattern in all samples, but trees grown at the wettest site (WL) often had a higher proportion of very small (<20 μm) vessels (Figure 2). Growth and thus DBH and ring width were highest at the wet and lowest at the dry site (Table 2).

**TABLE 2:** Mean (SD) of wood anatomical parameters, wood density and tree size per site and provenance.

Trait	VA	maxVA	Vcv	VD	VF	Ks	p.macro	Ks.micro	WD	DBH
Unit	μm^2^	μm^2^		mm^–1^		kg m^–1^ MPa^–1^ s^–1^	%	%	g cm^–3^	cm
**Site**										
Dry	2734 (794)	15453 (3154)	2.31 (0.40)	46.6 (9.7)	0.124 (0.031)	96 (40)	84.2 (5.7)	1.3 (1.0)	0.61 (0.05)	4.50 (1.05)
Interm.	2579 (617)	15753 (3351)	2.38 (0.43)	47.7 (12.4)	0.119 (0.029)	94 (39)	82.5 (4.5)	1.5 (0.7)	0.57 (0.02)	4.96 (1.24)
Wet	2498 (771)	16981 (3128)	2.60 (0.51)	47.1 (13.9)	0.110 (0.021)	93 (33)	81.3 (5.3)	1.4 (0.7)	0.56 (0.02)	6.68 (1.55)
**Provenance**										
1	2454 (610)	15921 (3208)	2.53 (0.44)	45.3 (7.3)	0.109 (0.023)	89 (36)	81.7 (4.8)	1.5 (0.7)	0.56 (0.02)	5.56 (1.48)
2	2367 (498)	17097 (3261)	2.62 (0.44)	49.7 (11.1)	0.115 (0.021)	95 (29)	81.3 (4.4)	1.4 (0.8)	0.58 (0.03)	5.99 (1.67)
6	2743 (754)	16276 (2579)	2.42 (0.48)	48.9 (16.7)	0.126 (0.034)	106 (48)	83.4 (5.6)	1.2 (0.6)	0.58 (0.04)	5.24 (1.37)
8	2820 (885)	15182 (2451)	2.25 (0.46)	41.4 (12.1)	0.110 (0.024)	81 (23)	84.5 (5.7)	1.2 (0.6)	0.58 (0.03)	4.85 (1.45)
12	2882 (949)	16699 (3557)	2.40 (0.51)	48.0 (13.2)	0.130 (0.027)	111 (39)	84.3 (4.6)	1.1 (0.5)	0.58 (0.05)	5.89 (1.73)
14	2475 (781)	17619 (3878)	2.65 (0.51)	51.0 (14.9)	0.120 (0.032)	104 (41)	82.5 (5.8)	1.2 (0.7)	0.59 (0.06)	5.74 (1.74)
17	2292 (584)	15511 (2848)	2.49 (0.32)	46.0 (8.9)	0.102 (0.018)	77 (26)	80.1 (5.7)	1.7 (1.2)	0.57 (0.03)	5.24 (1.29)
18	2457 (624)	14866 (3483)	2.34 (0.46)	46.1 (9.3)	0.110 (0.024)	79 (28)	81.4 (5.5)	1.8 (1.2)	0.58 (0.04)	5.04 (1.41)
19	2788 (835)	15384 (2814)	2.29 (0.44)	48.1 (14.0)	0.126 (0.027)	96 (32)	83.7 (5.0)	1.4 (0.8)	0.59 (0.07)	5.11 (1.45)
21	2760 (505)	16068 (3725)	2.29 (0.42)	46.9 (9.2)	0.130 (0.032)	105 (49)	83.7 (4.6)	1.4 (0.8)	0.57 (0.04)	5.18 (2.05)

*VA, mean vessel area; VAmp, mean area of macropores (vessels with diameter > 70 μm); VD, vessel density per cross-sectional area; VF, vessel lumen fraction; Ks, theoretical hydraulic conductivity; p_macro_, fraction of vessel area occupied by vessels with diameter > 70 μm; Ks_micro_, proportion of hydraulic conductivity contributed by vessels < 70 μm diameter; Vcv, coefficient of variation of vessel area; WD, wood density; DBH, diameter at breast height.*

**FIGURE 3:**
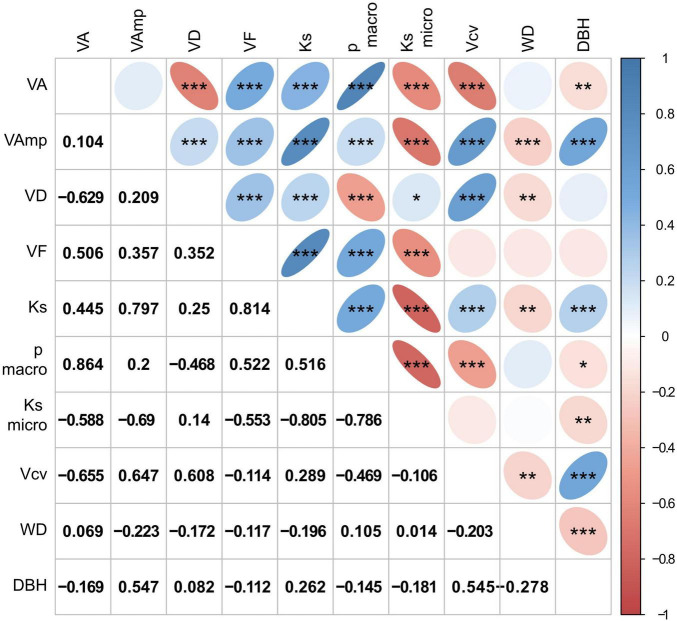
Correlation matrix of wood traits and tree size (DBH). Size and color intensity indicate strength of positive or negative correlations, blue for positive and red for negative correlations. Trait abbreviations as in Table 2. Significances in the upper triangle are indicated as *p < 0.05, **p < 0.01, ***p < 0.001, the lower triangle shows *r*-values.

Mean vessel size (VA) was positively correlated with VF, Ks, and the proportion of large vessels and negatively with VD, Ks_micro_, vessel size variation (Vcv), and DBH, but was not related to macropores size (VAmp, Figure 3). Ks is strongly controlled by VAmp and less by VA and VD, which is explained by the fact that Ks scales to the square of VA and the contribution of micropores (Ks_micro_) is low. The proportion of Ks contributed by small vessels was small, on average 2% and never more than 10%. Ks_micro_ scaled negatively with Ks calculated form all vessels and with VA and VAmp. Relationships with WD were weak and significantly negative for VAmp, VD, Ks, and Vcv. While the VAmp, Ks, and Vcv increased with tree size, the average vessel size, the proportion of large vessels and WD decreased with DBH. Correlations between wood traits were similar among sites, though correlations between anatomical traits and WD were significantly only for the intermediate and wet, but not the dry site (Supplementary Figure 2).

**FIGURE 4:**
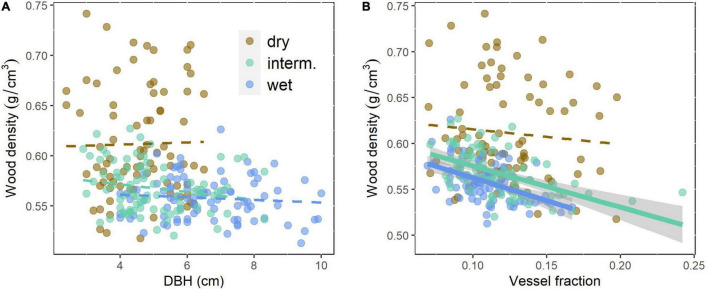
Wood density related to **(A)** tree size (DBH) as a measure of growth rate of the even-aged trees and **(B)** vessel fraction of *Quercus robur* at three trial sites differing in rainfall. Continuous lines indicate significant (*p* < 0.05) correlations, broken lines non-significant relationships. Site details are shown in Table 1.

VA, VF, WD, Ks, and p_macro_ decreased from the driest to the wettest site, while VAmp increased from the driest to the wettest site (Table 2). All vessel traits were explained to a larger extent by provenance (4.6–12.1% of the variance explained, Model 1) than by site (0.1–6.9%, Table 3). This contrasts with WD, which was explained strongly by site (30.7% explained) and was the only trait with significant provenance × site interactions. While overall WD is significantly negatively correlated with tree size, this is entirely due to a site effect and within site WD did not depend on tree size: Trees at the drier site were smaller and had higher WD. Within any site, WD and DBH were not correlated (*p* > 0.05, Figure 4A), but WD was related to VF at two sites (Figure 4B).

**TABLE 3:** Significance of provenance, site, tree size (DBH), and provenance × site interactions on wood traits.

	*p*-values				Variance explained (%)				
	Prov.	Site	DBH	Prov. × Site	Prov.	Site	DBH	Prov. × Site	Residual
VA	0.014	0.075	0.122	0.874	7.7	1.9	0.9	4.1	85.5
VAmp	0.004	4.8E-04	1.4E-18	0.796	6.5	4.1	23.9	3.3	62.2
VD	0.197	0.891	0.211	0.681	4.6	0.1	0.6	5.5	89.2
VF	4.5E-05	0.007	0.636	0.117	12.1	3.2	0.1	8.2	76.4
Ks	0.001	0.911	2.0E-06	0.436	9.4	0.1	7.7	5.9	76.9
p.macro	6.5E-04	2.0E-05	9.9E-10	0.718	8.8	6.5	11.7	4.1	68.9
Ks.micro	0.019	0.013	1.6E-06	0.350	6.6	2.8	7.8	6.4	76.5
Vcv	3.2E-04	3.7E-06	2.9E-15	0.803	8.5	6.9	18.7	3.3	62.6
WD	0.136	8.5E-23	0.208	5.0E-05	3.3	30.7	0.4	13.2	52.5

*Trait abbreviations as in Table 2. Shading from light to dark yellow indicates increasing significance.*

When the model included site and provenance, tree size did not affect VA, VD, and VF, but had a strong effect on maximum vessel size and consequently on Ks, Vcv, the proportion of macropores and Ks_micro_ (7.7–23.9% explained, Table 3). Model 2 with MAPp instead of provenance (Supplementary Table 1) shows that only a small part of the provenance effect can be explained by the differences in rainfall. Including DBH and site, MAPp was only marginally significantly related to VA, VF, and Vcv, and for none of these explained more than 1.5% of the variation.

When wood anatomy was analyzed year-wise with ring width and tree size included in the model (Model 3), provenance still had significant effects on VD and VF and a marginally significant (*p* < 0.1) effect on p_macro_ and Ks_micro_, but site effects were no longer significant and VF was the only parameter related to October–September precipitation (Table 4a). Ring width had a much stronger effect on nearly all wood traits, except for VAmp (Table 4b). Therefore, the site and to a large extent also the provenance effect in Table 3 are largely the result of differences in ring widths.

**TABLE 4:** (a) Significance of provenance, site, tree size (DBH), ring width, and precipitation (Prec is the precipitation from October of the previous year to September in the year the wood was produced) on vessel traits. Shading from light to dark yellow indicates increasing significance. For metric variables, the estimate and standard error (SE) of the estimate are also shown. **(b)** Shows the variance explained (%). Trait abbreviations as in Table 2.

(a)	Prov.	Site	Site ×	RW			DBH			Precip.		
			Prov.	*p*	Est	SE	*p*	Est	SE	*p*	Est	SE
VA	0.026	0.238	0.931	5.0E-15	−0.084	1E-03	0.968	−0.001	0.010	0.071	0.0147	4E-04
VAmp	0.568	0.827	0.632	1.1E-05	−5E-04	−6E-06	5E-19	0.001	1E-04	0.402	0.0002	6E-06
VD	0.439	0.666	0.612	6.8E-06	−0.040	−5E-04	1.4E-04	0.054	0.009	0.291	0.0141	4E-04
VF	0.009	0.397	0.167	5.9E-57	−0.126	5E-04	6.0E-06	0.047	0.007	0.016	0.0103	2E-04
Ks	0.245	0.843	0.491	3.4E-30	−0.154	3E-04	3.0E-12	0.139	0.013	0.619	0.0192	5E-04
p.macro	0.079	0.219	0.809	2.1E-07	−0.015	3E-04	0.63790681	0.002	0.003	0.084	0.0039	1E-04
Ks.micro	0.099	0.482	0.537	9.6E-07	0.096	−4E-04	2.0E-06	−0.131	0.019	0.561	0.0270	7E-04

(b)	Prov.	Site	Site × Prov.	RW	DBH	Prec.	Residual
VA	4.1	0.8	2.1	13.5	0.0	1.1	80.5
VAmp	1.1	0.1	2.2	2.8	13.3	0.1	82.6
VD	1.6	0.2	2.8	3.7	2.7	0.3	91.6
VF	2.2	0.3	2.4	30.0	2.1	1.2	64.2
Ks	1.3	0.0	1.9	15.8	5.9	0.0	77.0
p.macro	5.1	1.1	4.1	8.9	0.1	1.5	83.4
Ks.micro	3.0	0.3	3.4	5.0	4.8	0.1	86.8

**FIGURE 5:**
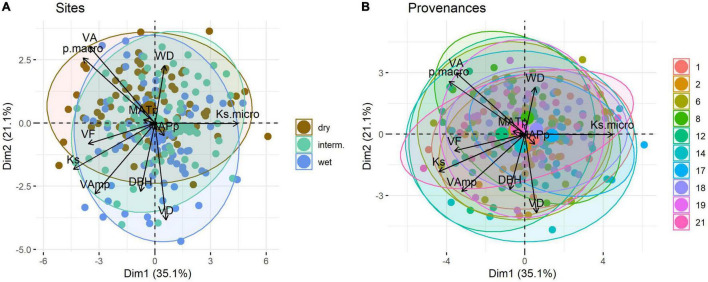
Principal component analysis of wood traits of *Quercus robur* grown at three trial sites in Austria **(A)** and originating from ten provenances **(B)**. Large symbols represent the mean position of trees per trial site or provenance along PC1 and PC2. Ellipse defines the region that contains 95% of all samples per group. Provenances are detailed in Table 1, trait abbreviations as in Table 2.

In the PCA Ks, Ks_micro_, p_macro_, VA, and VF scale along the first axis, which explains 35.1% of the variation, whereas VAmp, VD, WD, and DBH scale mainly along the second axis (21.1%) (Figure 5). MAPp and MATp had a very weak loading on the first two principal components. Trial sites separated poorly in the PCA (Figure 5A), as did provenances (Figure 5B).

**FIGURE 6:**
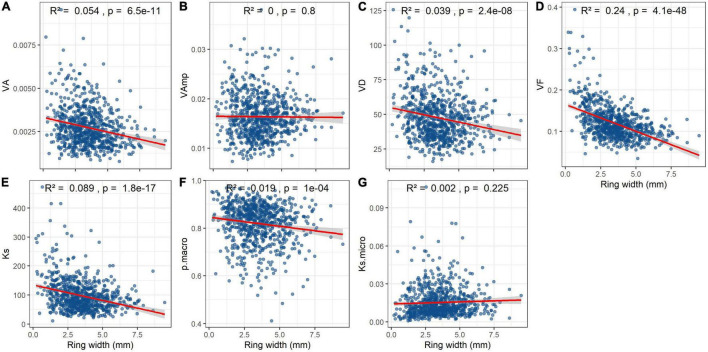
Effect of annual ring width on vessel parameters and hydraulic conductivity in *Quercus robur* wood. Trait abbreviations as in Table 2. Note that Ks is the theoretical hydraulic conductivity per cross-sectional area, whereas Ks_micro_ is the proportion of Ks that is contributed by vessels <70 μm. *R*^2^ and *p*-values are from linear regressions.

Mean VA, VD, Ks, and particularly VF significantly declined with ring width (Figures 6A,C–E). Macropore size and Ks_micro_ were not related to ring width at all (Figures 6B,F) and the relationship between p_macro_ and ring width weak (Figure 6F).

## Discussion

The effect of the environment on wood traits can be studied in various ways. Common garden experiments search for a genetic component in the variation by keeping the environmental variation constant. If this variation is related to a clinal gradient of their original locations, this trait variation may point to (but is not proof of) a potential genetic adaptation to a specific environmental factor. Experiments that expose different genotypes or provenances to different environments in a fully crossed design serve to quantitatively distinguish genetic from environmental effects. These experimental approaches are quite different from tree ring studies where inter- (and sometimes intra-) annual variations in climate are related to anatomical variation within individuals. This is ideal for dendroclimatology but does not study genetic adaptation (unless comparing different genotypes). It is also difficult to distinguish ontogenetic adaptations to short-term variations in the environment and direct or indirect effects of tree growth.

Our dataset is from a fully crossed trial with ten provenance and three trial sites (Table 1). Trees were 12 years old, so the wood is juvenile wood and wood in older trees might respond differently. Although several *Quercus* provenance trials are conducted (Gea-Izquierdo et al., 2012; Torres-Ruiz et al., 2019; Bert et al., 2020) and macropore size variation of *Quercus* has been studied in several tree ring analyses (e.g., Fonti and García-González, 2008; Martínez-Sancho et al., 2017), we are not aware of comparable studies in neither mature nor juvenile oak. With only 3 years of annual growth rings, our study is not suited to study short-term environmental effects. However, we use ring width as a control variable to test to which extent variation in wood anatomy may be a passive effect of variation in growth, which is important to understand and discuss any adaptive significance of anatomical variation. This may be particularly relevant in ring-porous *Quercus* where earlywood and latewood differ substantially in anatomy and the proportion of latewood mostly increases with ring width (Vavrčík and Gryc, 2012). The selection of provenances and trial sites for this study was designed to test for the effect of water supply on *Quercus* anatomy, and we had therefore chosen sites with a substantial rainfall gradient, but with a small temperature gradient.

Overall, vessel traits were strongly correlated (Figure 3). This is to be expected given the strong correlation between VD and VA commonly observed across (Zanne et al., 2010) but also within species (Rungwattana and Hietz, 2018) and the fact that the other anatomical traits are in one way or another derived from VA and VD. WD, while correlating significantly with several anatomical parameters never explained more than 5% (for VAmp) of their variation.

### Wood Density Variation

The control of the intra-specific variation in WD, which is of substantial practical importance for the timber industry, has been extensively studied (Zobel and van Buijtenen, 1989). WD variation is relatively well-understood and studies have shown a significant inheritance (Zobel and Jett, 1995). By contrast, we found the site effect (explaining 30.7% of the variation) much higher than the provenance effect, with significant site × provenance interaction (Table 3). Ring-porous trees including *Quercus robur* and *Q. petraea* (Zhang et al., 1993) tend to have higher WD when they are growing fast. In these trees, the thickness of earlywood, with large vessels and low density, is less variable, when growth rates are high more latewood with small vessels and high WD is added (Saranpää, 2003). This was not reported in all studies and site conditions may or may not additionally affect WD (Zobel and van Buijtenen, 1989 and many older studies cited therein). If WD was controlled by the early wood or late wood ratio and late wood had higher WD, WD should scale positively with growth rates. Surprisingly, we found WD to be negatively correlated with tree size, but this was entirely a site effect with trees at the drier site being smaller and having higher WD. Within any of the three sites, there was no correlation (Figure 4A). WD will be affected by vessel lumen fraction because lumina, unless filled with extractives, effectively have a density of 0. VF did explain a substantial part of the variation in WD in the intermediate and wet sites (Figure 4B), but not in the dry site and does not explain the difference in WD between sites. In many tree species, WD increases or less commonly decreases with tree size (Hietz et al., 2013), the latter has been observed in *Quercus* (Zhang et al., 1993). Other studies suggest that some size-related changes in wood characteristics may actually be controlled by cambial age rather than tree size (Lachenbruch et al., 2011). None of this can explain the variation between sites observed as all trees were of the same age and WD was higher in the dry site independent of tree size (Figure 4A). Most studies that looked into the anatomical basis of WD variation found that this is mostly achieved by the variation in fibers and particularly fiber wall thickness (Martínez-Cabrera et al., 2009; Ziemińska et al., 2014). High WD and fibers are clearly important for the mechanical strength of wood, and across species, WD explains c. 80% of the variation in various measures of mechanical strength (Niklas and Spatz, 2010). However, there is no obvious explanation as to why trees in drier sites would need to be mechanically stronger.

Alternatively, WD may be related to drought tolerance, whether in some way affecting cavitation resistance or otherwise. Some studies found a moderately significant link between WD and cavitation resistance (Awad et al., 2010; Fichot et al., 2010; Guet et al., 2015) within species. Many studies have also reported higher WD in dry regions, although across species aridity explains only a small part in the variation of WD (Ibanez et al., 2017). Lacking an alternative explanation for the variation of WD, our study lends cautious support to the idea that high WD is an adaptation to drought. However, we note that this variation is explained by plastic and not by genetic adaptation, which means that selecting drought-tolerant individuals or provenances based on the WD from the provenance site is not a workable strategy.

### Wood Anatomical Variation

The control of WD contrasts with wood anatomical parameters, which were primarily under provenance (i.e., genetic) control with less site effects and none for Ks (Tables 3, 4). Interestingly, tree size, which has been shown to affect wood anatomical parameters, had no significant effect on mean VA, VD, and VF, but does affect macropores and consequently Ks and parameters related to vessel size variation (Table 3). The importance of tree size on wood structure is well-known, and more recent studies are including this effect in studies that try to understand the adaptive value of wood anatomy (Hietz et al., 2017; Rosell et al., 2017). Accounting for tree size, we found significant site and/or provenance effects on most measures. However, studying the annual resolution and including ring width and tree size, site effects completely disappear and provenance effects are weaker. This suggests that site and to a large extent provenance effects mainly result from the effects on growth and thus ring width. Except for VAmp, also the tree size effect is largely the result of variations in ring width (Table 4 and Figure 6), likely through the variation in the proportions of earlywood and latewood. Ring-porous trees such as *Quercus robur* show strong differences in wood anatomy between early- and latewood, the proportion of which is affected by ring width. We expect that the dominant effect of ring width or growth rates seen here will be less in diffuse-porous species and perhaps absent in tropical trees that have less annual variation in wood structure.

When tree size and ring width are included, only VF was significantly related to rainfall (Table 4). Since this analysis relates wood parameters to rainfall at the trial sites in the year the wood was produced plus rain since the previous October, we are here testing for plastic adaptations. We are aware that this analysis is less than ideal as there are only 9 years (3 years at three trial sites) to test, for which reason we refrain from testing for effects of rainfall in individual months or seasonal climate variation as is common in dendroclimatological studies.

Average VA and Ks (though not VAmp) were greater in trees growing in the drier sites. This appears surprising given that these trees are smaller and thus need to supply fewer leaves and may have less water available to transport. Similarly, Pérez-de-Lis et al. (2018) found larger vessels in *Q. robur* and *Q. pyrenaica* from drier regions and speculate about the potential benefit of high hydraulic conductivity in trees from seasonal climates. However, using Ks here is misleading. In oaks as in other ring-porous trees, only the outermost ring(s) typically conduct water (Poyatos et al., 2007). The capacity of a stem to transport water is the hydraulic conductivity (Ks) times the conductive area. Therefore, if the same number of outer rings contributes to water transport, the potential conductance should be approximately proportional to conductivity x ring width. Average ring width was greater in the wet (4.3 mm) than in intermediate (3.3) and dry (2.8) site, thus the hydraulic conductance of the whole stem is likely greater in the wetter site and speculations about the advantage of high conductivity in dry sites are unfounded. Similarly, the larger mean vessel areas at the dry site can be explained by the fact that the thinner annual growth rings had proportionally less latewood and thus a greater proportion of macropores (Table 2).

Various hydraulic traits including xylem vulnerability to cavitation, leaf turgor loss point, and osmotic potential (Pfautsch et al., 2016; Li et al., 2018; Kunert et al., 2021) have been shown to correlate with indices of aridity across species, sampled at their natural environment or a common garden. Within species, the trait variation is generally smaller and their climatic range is more limited, so finding significant trends is more challenging. Indeed, there are few studies, at least for hardwoods, directly addressing adaptations of intra-species variation in wood anatomy. Hajek et al. (2016) found vessel diameter (but not VD) of *Fagus sylvatica* grown in a common garden and originating from ten locations across Europe significantly related to the forest aridity index at their place of origin. However, in their dataset, the correlation with mean annual temperature was almost as high and no attempt was made to distinguish between adaptations to temperature vs. aridity. Another study found that the xylem was more resistant to cavitation in Mediterranean compared to Central European *Quercus* species, but there was no difference between provenances within *Q. robur* (Lobo et al., 2018). In this study, we only tested for clines related to MAPp because there were only ten provenances. We did not try to correlate a host of different aridity measures with a limited dataset, which is likely to produce spurious correlations, but we are aware that MAP may not be the best measure of aridity.

The overall pattern of vessel size variation was remarkably constant across sites and provenances and showed a peak for small vessels with a maximum density at c. 20 μm diameter and a much smaller peak for vessels > 70 μm diameter (Figure 2). Like other anatomical parameters, vessel size variation, the fraction occupied by large vessels and the proportion of hydraulic conductivity contributed by small vessels was controlled largely by tree size, ring width and provenance, and only to a minor extent by site. While most vessel parameters except for VAmp were related to ring width (Figure 6 and Table 4), the effect was very small for p_macro_ and Ks_micro_. Vessels < 70 μm diameter in most cases contributed <3% to total Ks. Although Ks_micro_ showed a significant effect of provenance, it was not related to the climate at the provenance location (Table 4). Small vessels contribute less to Ks but might still be relevant when large vessels are embolized, and wood needs to transport a minimum of water to leaves with strongly reduced stomatal conductance as a consequence of drought. However, we found no evidence that the contribution of small pores would increase by plastic or genetic adaptation to aridity in *Q. robur*.

According to the most accepted model, xylem emboli arise under drought stress when the pressure gradient across the cell wall permits gas to pass through the pit membranes. Emboli can also form when after freeze-thaw events minute gas bubbles coalesce and expand and water tension is high enough. In the latter case, the vulnerability is directly related to vessel size (large vessels thawing yield more and larger gas bubbles), whereas in the first case, vulnerability is related to the fine structure of the cell walls and only indirectly to vessel size (rare pith hypothesis). Indeed, experimental studies found a stronger link between vessel size and loss of conductivity following freezing (Davis et al., 1999) than between vessel size and drought-induced cavitation (Jacobsen et al., 2019). A global analysis of woody plants found small vessels to be an important adaptation to cold environments (Zanne et al., 2014). This suggests that it would be worthwhile to investigate intra-specific variation in vessel size also in relationship with minimum temperature and not mainly focus on adaptations to drought. That said, unless there is clearer evidence for a climate signal carried by small vessel, the substantially greater effort to measure these may not justify the additional time that would be needed for the studies in dendroclimatology.

Other provenance studies found particularly phenology under strong genetic control and more so than leaf traits or WD. A stronger genetic control for leaf than for wood traits was also shown in a previous paper on the *Q. robur* provenance trial (George et al., 2020). This study found the genetic control of WD (i.e., the provenance effect) among the lowest of all wood traits measured (Table 3). This is important for tree breeding programs that seek to select genotypes better adapted to a warmer climate with higher water deficit. However, we also caution against quick recommendations based on the individual traits and correlations with climate. First, while drought resistance (at least xylem vulnerability, which is only one component of drought resistance) has been shown to correlate and to some extent is functionally related with wood traits (see Section “Introduction”), these relationships are often quite weak. Second, while anatomical traits may be under strong genetic control and the result of evolutionary selection, the relationship to climate is often weak. Third, the provenance trials that have looked at wood traits other than WD mostly include temperature as well as aridity clines (the latter often weaker) in the climate of origin, which makes it difficult to distinguish between adaptations to temperature or aridity. Finally, wood traits represent only part of the complex adaptations to drought. Adjustments in roots, leaves, phenology, or other components can be equally or more important (e.g., Rungwattana et al., 2018). A study on 22 North American tree species found that, while WD was positively related to cavitation resistance, species with high WD actually suffered more under drought whereas species with low WD suffered less as these relied on various strategies of drought avoidance (Hoffmann et al., 2011). Similarly, deciduous trees in tropical dry forests follow quite different strategies (high hydraulic conductivity, low WD, high water storage, and high xylem vulnerability) to survive under similar conditions as evergreen trees (Méndez-Alonzo et al., 2012). The ultimate measure for the trees’ adaptations to and suitability for a given environment should be growth and mortality. In the *Q. robur*, provenance trial trees grew better if transferred to a climate similar to their provenance (George et al., 2020). This effect was also stronger for temperature than for precipitation. More complex datasets and provenance trials should try to disentangle effects of and adaptations to variation in water availability as well as temperature but need to account for tree size or growth rates to avoid a biased interpretation of trait adaptations.

## Data Availability Statement

The raw data supporting the conclusions of this article will be made available by the authors, without undue reservation.

## Author Contributions

PH conceived the idea, analyzed the data, and wrote the manuscript. KR and SS sampled wood and analyzed wood samples. J-PG sampled wood and provided access to and additional information on the provenance trial. All authors contributed to the article and approved the submitted version.

## Conflict of Interest

The authors declare that the research was conducted in the absence of any commercial or financial relationships that could be construed as a potential conflict of interest.

## Publisher’s Note

All claims expressed in this article are solely those of the authors and do not necessarily represent those of their affiliated organizations, or those of the publisher, the editors and the reviewers. Any product that may be evaluated in this article, or claim that may be made by its manufacturer, is not guaranteed or endorsed by the publisher.

## Footnotes

1
imagej.nih.gov/ij

2
www.zamg.ac.at
