# Supplementary material for: Effects of Provenance, Growing Site, and Growth on Quercus robur Wood Anatomy and Density in a 12-Year-Old Provenance Trial
Source: Front Plant Sci. 2022 Apr 29;13:795941. doi: 10.3389/fpls.2022.795941 (PMC9100569; doi:10.3389/fpls.2022.795941)
Supplement: Supplementary file 1 [file Table_1.DOCX]

Supplementary Material

Effects of provenance, growing site and growth on *Quercus robur* wood anatomy and density in a 12-year-old provenance trial

**Peter Hietz, Kanin Rungwattana, Susanne Scheffknecht, Jan-Peter George**

|  |  | p-values |  |  |  |  |  | Variance explained (%) |  |  |
| --- | --- | --- | --- | --- | --- | --- | --- | --- | --- | --- |
|  | MAPp | Site | DBH | MAP x Site |  | MAPp | Site | DBH | MAPp x Site | Residual |
| VA | 0.055 | 0.075 | 0.065 | 0.172 |  | 1.3 | 1.9 | 1.2 | 1.3 | 94.3 |
| VAmp | 0.677 | 4.2E-04 | 1.6E-21 | 0.432 |  | 0.0 | 4.1 | 27.9 | 0.4 | 67.5 |
| VD | 0.602 | 0.890 | 0.063 | 0.116 |  | 0.1 | 0.1 | 1.3 | 1.6 | 96.9 |
| VF | 0.086 | 0.013 | 0.898 | 0.360 |  | 1.1 | 3.2 | 0.0 | 0.7 | 95.0 |
| Ks | 0.266 | 0.915 | 2.0E-07 | 0.247 |  | 0.4 | 0.1 | 9.6 | 1.0 | 88.9 |
| p.macro | 0.177 | 0.001 | 0.606836128 | 0.132 |  | 0.6 | 4.8 | 0.1 | 1.4 | 93.1 |
| Ks.micro | 0.305 | 0.015 | 5.8E-07 | 0.080 |  | 0.3 | 2.8 | 8.7 | 1.7 | 86.5 |
| Vcv | 0.073 | 2.8E-06 | 1.8E-18 | 0.059 |  | 0.8 | 6.9 | 23.0 | 1.5 | 67.7 |
| WD | 0.229 | 3.7E-20 | 0.370 | 0.160 |  | 0.4 | 30.1 | 0.2 | 1.0 | 68.3 |

**Table S1.** Effect of MAP at provenance, site, tree size (DBH) and provenance x site interactions on wood traits. Trait abbreviations as in Fig. 2.


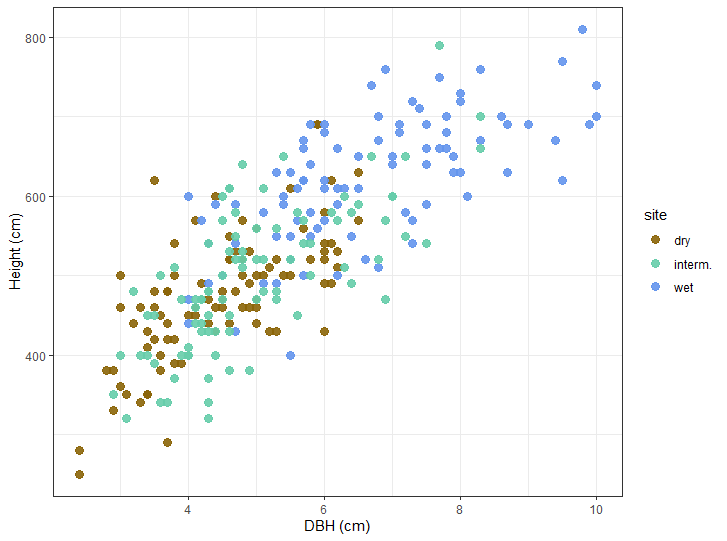


Fig. S1. Relationship between tree diameter (BHD at 10 years) and height (H) for sampled trees from the three trial sites.

Intermediate site


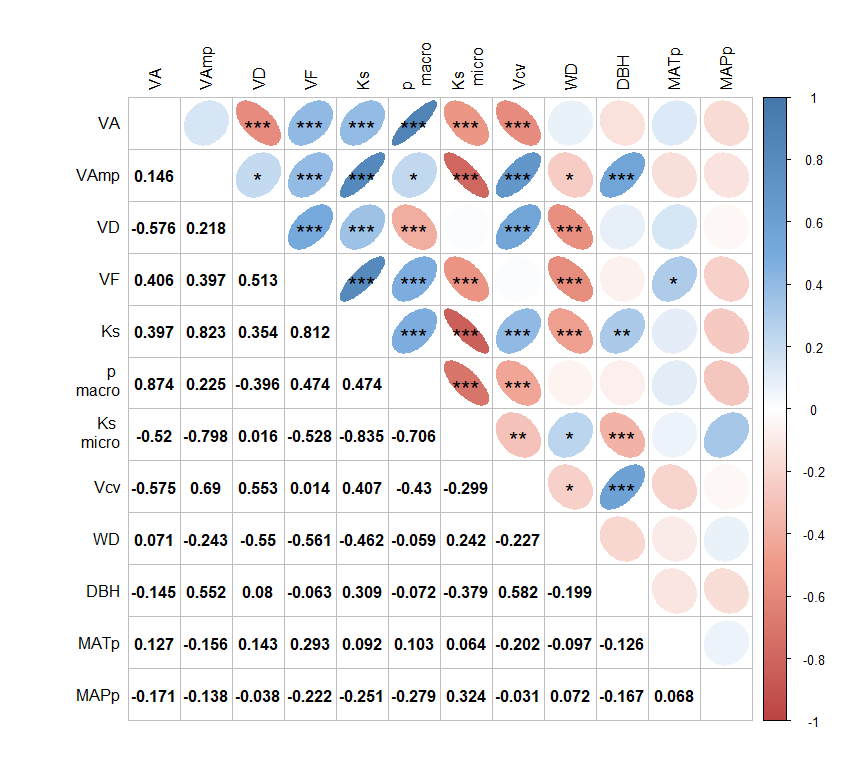


Dry site


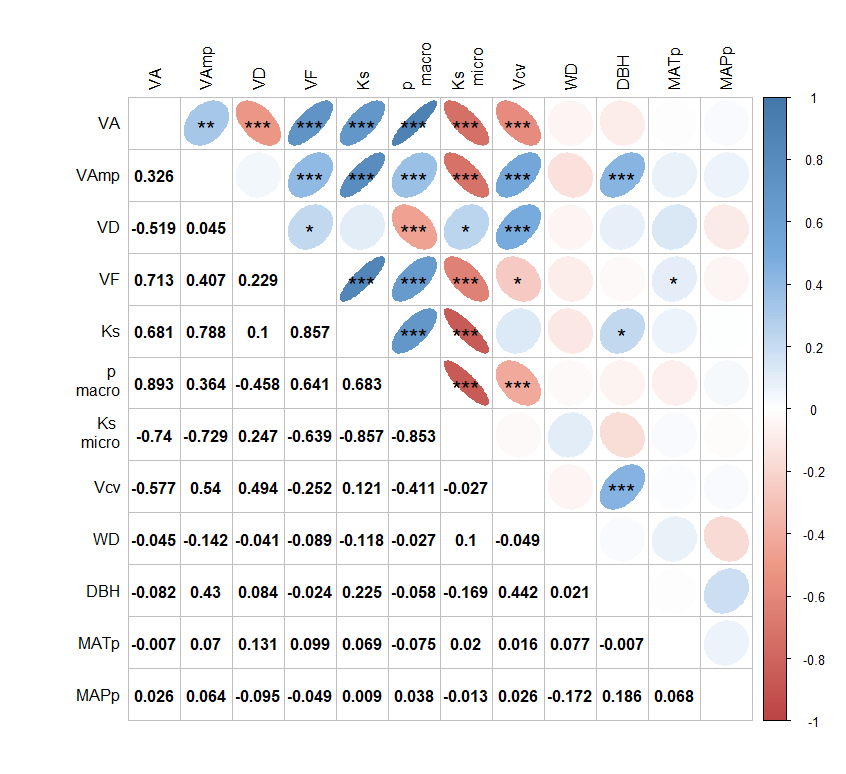

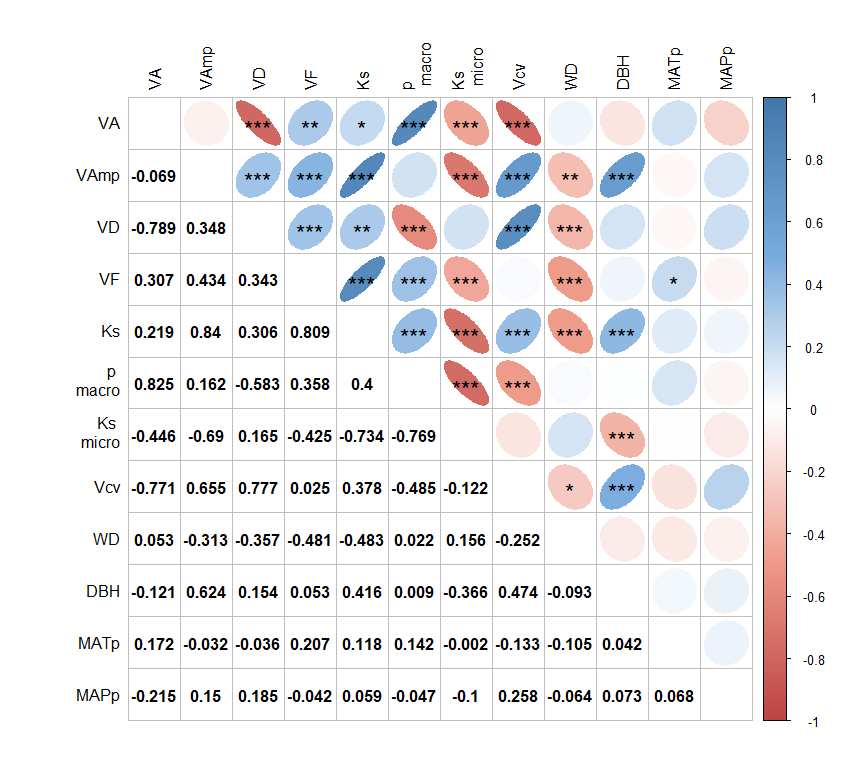


Wet site

**Fig. S2.** Correlation matrix of wood traits and tree size (DBH), as Fig. 3 but separate for the three sites. Size and colour intensity indicate strength of positive or negative correlations, blue for positive and red for negative correlations. Trait abbreviations as in Table 2. Significances in the upper triangle are indicated as *: p < 0.05, **: p < 0.01, *** : p < 0.001, the lower triangle shows r-values.
